# Investigating dysfunctional cognition change as a putative mechanism of CBT for youth anxiety, OCD and PTSD: protocol for an individual participant data meta-analysis

**DOI:** 10.1136/bmjopen-2025-113007

**Published:** 2025-12-03

**Authors:** Ivana Buric, Anke Klein, Ronald M Rapee, Brooke Levis, Philip C Kendall, Eric A Storch, Lynn Mobach

**Affiliations:** 1 Department of Psychology, University of Amsterdam, Amsterdam, The Netherlands; 2 Developmental and Educational Psychology, Leiden University, Leiden, The Netherlands; 3 Lifespan Health and Wellbeing Research Centre, Macquarie University, Sydney, New South Wales, Australia; 4 Department of Epidemiology, Biostatistics and Occupational Health, McGill University, Montreal, Quebec, Canada; 5 Department of Psychology and Neuroscience, Temple University, Philadelphia, Pennsylvania, USA; 6 Department of Psychiatry and Behavioral Sciences, Baylor College of Medicine, Houston, Texas, USA; 7 Institute for Integrated Mental Health Care, Pro Persona, Overwaal, Nijmegen, The Netherlands

**Keywords:** Child & adolescent psychiatry, Meta-Analysis, Cognitive dysfunction, MENTAL HEALTH

## Abstract

**Abstract:**

**Introduction:**

Anxiety disorders, obsessive–compulsive disorder (OCD) and post-traumatic stress disorder (PTSD) are common in children and adolescents and can lead to significant impairment. Cognitive behavioural therapy (CBT) with exposure is the first-line treatment, yet approximately half of treated youth do not achieve full remission. Dysfunctional cognitions—negative automatic thoughts, maladaptive beliefs and distorted interpretations—are considered key targets of CBT, but evidence in youth is mixed and underpowered. This study will examine whether change in dysfunctional cognitions mediates treatment outcome in anxiety, OCD and PTSD symptoms and whether this association varies across individual characteristics.

**Methods and analysis:**

An individual participant data meta-analysis (IPDMA) of randomised controlled trials of CBT for youth aged 5–18 years with anxiety disorders, OCD or PTSD will be conducted. The search strategy includes the databases APA PsycINFO, MEDLINE and Web of Science Core Collection from inception to 8 September 2025. It is supplemented by screening reference lists, trial registries, grey literature and outreach to relevant research groups. Eligible trials must include at least one validated measure of dysfunctional cognitions administered at minimum pre- and post-treatment, and clinical outcomes assessed at post-treatment and follow-up. The two primary outcomes are (1) child-reported symptom severity and (2) clinician-rated clinical severity. Data will be harmonised for dysfunctional cognition scores, moderators (age, gender, socioeconomic status, comorbidity), and primary outcomes. One-stage Bayesian mixed-effects models will examine whether changes in dysfunctional cognitions predict improvements in primary outcomes and whether these effects are moderated by individual characteristics. Missing data will be addressed using multiple imputation within the Bayesian framework, and study-level heterogeneity will be modelled using random intercepts and slopes.

**Ethics and dissemination:**

All datasets will be de-identified and managed under General Data Protection Regulation standards. Each included trial will have ethical approval permitting data sharing and reuse, and the secondary analysis of the shared datasets has been approved by the University of Amsterdam. Findings will be disseminated via a peer-reviewed publication, scientific conferences and open sharing of analysis scripts and harmonisation procedures.

**PROSPERO registration number:**

CRD420251139130.

**STRENGTHS AND LIMITATIONS OF THIS STUDY**

- A one-stage individual participant data meta-analysis (IPDMA) will use Bayesian mixed-effects models to analyse changes in dysfunctional cognitions during Cognitive behavioural therapy (CBT) for youth with anxiety disorders, obsessive–compulsive disorder (OCD) or post-traumatic stress disorder (PTSD).
- Combining data from multiple international trials provides greater statistical power to detect individual-level moderators and examine whether changes in dysfunctional cognitions predict treatment outcomes.
- Pooling individual participant data from multiple randomised controlled trials allows for uniform analyses and harmonisation of all variables.
- Representativeness and generalisability will depend on the availability, completeness and quality of shared datasets from eligible trials.

## Introduction

 Anxiety disorders, obsessive–compulsive disorder (OCD) and post-traumatic stress disorder (PTSD) are among the most common psychological difficulties in children and adolescents, with a lifetime prevalence ranging from 15% to 20%.^1^,^3^ These disorders are associated with impairment in social, academic and family functioning^4^,^7^ and they increase the likelihood of developing comorbid disorders such as depression and substance use.^8^ Despite their prevalence and associated burden, many of the affected youth do not receive appropriate treatment,^9^ and outcomes remain suboptimal even for those who do.^10^ Exposure-based Cognitive Behavioural Therapy (CBT) is the current gold-standard treatment for these conditions, yet approximately half of treated youth do not achieve sufficient symptom relief.^11^ Although the efficacy of CBT has been the focus of substantial research efforts, treatment effects have not meaningfully improved over the past four decades and show considerable variability across individuals.^11^ These findings have led to growing consensus that youth psychotherapy research must move beyond the question of ‘whether’ CBT works to a deeper understanding of ‘how’ it works and ‘for whom’ to improve individual outcomes of CBT (eg,^11^).

Within CBT, exposure and cognitive restructuring are regarded as the critical working elements for treating anxiety-related disorders, including OCD and PTSD.^12^,^15^ Theoretical models commonly assume that both exposure and cognitive restructuring work by correcting dysfunctional cognitions—maladaptive beliefs about threat, control or vulnerability that maintain anxiety and stress-related disorders.^1216^,^18^ However, this assumption has been systematically examined only in adults, where a growing body of evidence suggests change in dysfunctional cognitions as one of the key mechanisms of CBT.^19^ In contrast, empirical evidence for this mechanism in children and adolescents remains limited and inconsistent. Developmental factors, such as cognitive maturation or emotion regulation capacity, may influence how youth engage with exposure and cognitive restructuring and whether cognitive change plays a central mechanistic role.^20 21^ Although some studies have reported that reductions in dysfunctional cognitions are associated with symptom improvement in youth,^22^,^25^ other studies have found only partial support for this association.^26^,^29^

Methodological limitations, such as the predominance of uncontrolled or single-arm study designs, small sample sizes and inconsistencies in how dysfunctional cognitions are operationalised, may contribute to these mixed findings. Moreover, CBT outcomes in anxious youth show considerable heterogeneity, suggesting that CBT does not operate uniformly across all children. Individual characteristics such as age, gender, socioeconomic status and comorbidity may moderate treatment response and its underlying mechanisms.^30^ However, most youth psychotherapy trials are statistically underpowered to detect reliable mechanisms or moderators, especially when effect sizes are small to moderate,^11^ which increases the risk for type I and type II errors. Addressing these complex questions—whether dysfunctional cognition change during treatment predicts anxiety, OCD or PTSD complaints, and whether this relationship varies across subgroups—requires access to large, individual-level datasets.

Traditional meta-analyses using aggregate-level data are limited in their ability to test individual-level associations and are vulnerable to ecological bias and confounding factors.^31 32^ An individual participant data meta-analysis (IPDMA) offers a powerful and flexible alternative. By pooling de-identified participant-level data from multiple trials, IPDMA enables harmonisation of measures and covariates, uniform treatment of missing data and robust statistical modelling of participant-level predictors and associations with heightened power.^33 34^ Given the growing availability of trials including psychometrically sound measures of dysfunctional cognitions, an IPDMA is both timely and necessary to investigate for whom and how exposure-based CBT is most effective with greater precision and scope than traditional meta-analyses. By facilitating collaboration across research groups and transparency through data sharing,^34 35^ this approach can accelerate cumulative knowledge-building in youth psychotherapy, where individual trials are often too small and underpowered to identify moderators or mechanisms of treatment response.

To our knowledge, this study is the first IPDMA to investigate dysfunctional cognition change as a putative mechanism of CBT for youth anxiety, OCD and PTSD. The primary aim is to examine whether changes in dysfunctional cognitions during treatment predict reductions in two key clinical outcomes: child-reported symptom severity and clinician-rated clinical severity among youth aged 5–18 years. A secondary aim is to investigate whether the strength or direction of these associations varies by individual characteristics, including age, gender, socioeconomic status, comorbidity and baseline levels of cognitive distortions. By investigating putative mechanisms and individual-level moderators of CBT response, this study aims to generate evidence that can inform the tailoring of CBT to more effectively meet the needs of individual children and adolescents.

## Methods

### Eligibility criteria

Studies will be eligible for inclusion in this IPDMA if they meet the following criteria:

### Study design

Studies must be randomised controlled trials (RCTs), including cluster-RCTs and follow-up or secondary analyses of original RCTs, conducted in any setting. Trials must evaluate a CBT intervention and report on its therapeutic effects. Comparators may include passive controls (eg, waitlist), treatment as usual, other psychological interventions or pharmacological treatments.

### Population

Participants must be youth aged 5 to 18 years with a primary diagnosis of one of the following Diagnostic and Statistical Manual of Mental Disorders-based disorders: generalised anxiety disorder, separation anxiety disorder, social anxiety disorder, specific phobia, selective mutism, OCD or PTSD. Diagnoses must be established using a structured or semi-structured diagnostic interview (eg, Anxiety Disorders Interview Schedule). Studies targeting populations with comorbid conditions (eg, autism spectrum disorder, depression, attention deficit hyperactivity disorder) will be included if anxiety, OCD or PTSD is the primary focus of treatment. Trials exclusively targeting populations with chronic medical or neurodevelopmental disorders will be excluded.

### Outcomes

Studies must report outcomes related to anxiety, OCD or PTSD symptoms. Eligible outcomes include clinician-rated clinical symptom severity (eg, Clinical Severity Rating (CSR), or validated child-reported symptom severity measures. To permit longitudinal analyses of cognitive change, studies must include a minimum of three assessment timepoints (typically pre-treatment, post-treatment and follow-up) and must include at least one validated measure of dysfunctional cognitions administered at a minimum of two timepoints (pre-treatment and post-treatment). Eligible dysfunctional cognition measures are defined as instruments that assess negative automatic thoughts, maladaptive beliefs or distorted interpretations relevant to anxiety, OCD or PTSD in youth. Common examples include the Children’s Automatic Thoughts Scale, Child Post-Traumatic Cognitions Inventory or Children’s Negative Cognitive Errors Questionnaire.

### Language and publication status

Studies published in English, Dutch or German will be included. Both published and unpublished data will be considered, provided the trial meets all eligibility criteria and authors are willing to share individual participant data with appropriate ethical approvals.

### Patient and public involvement

A patient organisation was involved in shaping the design of this research to ensure its relevance and feasibility from the patient perspective. Ongoing involvement of both the patient organisation and a patient panel is planned throughout the conduct of the study, and they will also contribute to the reporting and dissemination of the findings.

### Study search and selection

This protocol adheres to the Preferred Reporting Items for Systematic Review and Meta-Analysis Protocols (PRISMA-P) guidelines.^36^ At the time of writing, the systematic search has been completed, and potentially eligible studies have been identified. Authors of these trials have been contacted to confirm eligibility and invite collaboration. Several trialists have already shared individual participant data, while others have indicated willingness to share data pending final eligibility checks (ie, the process of dataset acquisition is ongoing).

A systematic literature search was conducted on 5 April 2024 and updated on 8 September 2025 to identify randomised controlled trials evaluating CBT with an exposure component in youth with anxiety, OCD or PTSD. Searches were carried out in three electronic bibliographic databases: APA PsycINFO (via Ovid), MEDLINE (via Ovid) and Web of Science Core Collection. These databases were selected for their comprehensive coverage of psychological and medical research. Scopus and Embase were reviewed as potential additional sources but not formally searched due to substantial content overlap with the databases already included. The search strategy was developed in collaboration with an information specialist. It combined controlled vocabulary (eg, MeSH terms and subject headings) and free-text keywords for three core concepts: (1) dysfunctional cognitions (eg, automatic negative thoughts, distorted interpretations), (2) target populations (youth with anxiety, OCD or PTSD) and (3) age range (youth aged 5–18 years). The full search strategies for each database are provided in the online supplemental material. All search results were imported into Zotero reference management software and deduplicated. Two reviewers independently screened titles and abstracts. Full-text articles were retrieved for studies deemed potentially eligible, and inclusion decisions were made independently by both reviewers using prespecified eligibility criteria. Disagreements were resolved by consensus or, if needed, by a third reviewer. When eligibility cannot be confirmed based on published information, authors were contacted for clarification. Eligible trialists were invited to collaborate and asked to provide de-identified individual participant data (IPD) for all randomised participants, including data that may not have been reported in the published manuscripts. Studies will be included in the IPD meta-analysis only if the authors agree to share IPD and ethical approval for data sharing has been obtained or can be confirmed.

To supplement database searches, we will be screening reference lists of relevant systematic reviews and included studies. Grey literature will also be considered by scanning Google Scholar. Clinical trial registries will be searched and flagged for inclusion in future search updates if needed. In addition to contacting authors of eligible studies identified through database searches, we will also be reaching out to investigators from relevant research groups via email, professional networks and at scientific conferences to identify additional eligible trials.

### Data sharing procedure

We will be inviting principal investigators of eligible trials to join the collaboration and contribute de-identified IPD for all randomised participants, including data not reported in published manuscripts. An accompanying document will be provided detailing the list of requested variables and their definitions for inclusion in the merged collaborative dataset. These will include diagnostic status, symptom and dysfunctional cognition measures, demographic information (eg, age, gender, socioeconomic status), group allocation, and all relevant timepoints (eg, baseline, post-treatment and follow-ups). If no response is received from the principal investigator after three follow-up reminders, we will then contact all other co-authors listed on the publication.

Contributing sites will be encouraged to share a cleaned dataset containing only the requested variables. However, if this is not feasible, full datasets may also be shared, and our team will screen, extract and clean the relevant variables in consultation with the original investigators. Before joining the collaboration, contributing sites must ensure that appropriate ethical approvals are in place for the sharing of de-identified data for secondary analysis. Data should be submitted in SPSS or Excel formats and transferred securely via a General Data Protection Regulation (GDPR)-compliant platform SURF FileSender (https://filesender.surf.nl). Our IPDMA team will implement a reproducible data management pipeline to process, merge and harmonise datasets. All datasets will undergo systematic quality control focused on variables relevant to the IPDMA, including checks for completeness, coding consistency and missingness. Any discrepancies or uncertainties will be resolved in consultation with the original investigators.

Initial feedback on the proposed aims and analysis plan will be welcome from all collaborators. While the main analyses are pre-specified, the received data may also inform post-hoc or exploratory analyses that go beyond the original protocol. Contributing investigators will have the opportunity to review the harmonised dataset and variable coding before analysis and to participate in the interpretation and dissemination of findings, in line with the consortium’s agreed governance and authorship policies.

### Data management and harmonisation

All datasets received from contributing trialists will be processed and harmonised using a reproducible data management pipeline developed by the IPDMA team. Variable names, formats and coding schemes will be standardised across trials to allow for pooled analysis. Where necessary, construct-level harmonisation will be conducted to align semantically equivalent variables that are coded differently across studies.

Moderators of interest will be harmonised as follows: age will be retained as a continuous variable in years (converting months to years where needed); sex will be coded as a three-level categorical variable (male, female, other/non-binary) and comorbidity will be coded as a binary indicator of any additional psychiatric diagnosis based on structured clinical interviews. Socioeconomic status will be harmonised using a three-level categorical scheme (low, medium, high) prioritising parental income relative to local contexts when available. If income data are missing, harmonisation will rely on parental education or occupation using comparable thresholds.

Dysfunctional cognitions (negative automatic thoughts, maladaptive beliefs or distorted interpretations related to anxiety, OCD or PTSD) will be standardised using z-scores. If a study includes more than one validated measure of dysfunctional cognitions, standardised scores will be averaged to create a single composite score.

Treatment outcomes will be harmonised into two variables: (1) clinician-rated symptom severity and (2) child-reported symptom severity. Clinician-rated severity will be based on CSR derived from structured diagnostic interviews, which reflect symptom intensity on a dimensional scale from 0 to 8 (a CSR score of 4 or higher indicates the presence of a clinical diagnosis). Child-reported severity will be based on validated self-report measures of anxiety, OCD or PTSD symptoms. If multiple validated child self-report measures are available within a study, we will prioritise scales that most closely align with the primary diagnosis. Both outcomes will be standardised using z-scores. In sensitivity analyses, binary diagnostic status may also be examined, defined as 0 (no current diagnosis/remission) or 1 (diagnosis present), with remission based on a CSR below the clinical threshold (ie, CSR <4).

All harmonisation decisions will be documented in a codebook specifying original variable names, coding rules and the resulting harmonised variables. Internal cross-checks and visual inspection of distributions (eg, histograms and density plots) will be conducted to ensure the accuracy and consistency of harmonisation. Discrepancies or unexpected patterns will be resolved in consultation with the original trial teams.

### Risk of bias and meta-biases

Risk of bias will be independently assessed for each included study using the revised Cochrane risk-of-bias tool for randomised trials (RoB 2).^37^ This tool evaluates bias across five domains: (1) the randomisation process, (2) deviations from intended interventions, (3) missing outcome data, (4) measurement of the outcome and (5) selection of the reported result. Two reviewers will independently assess each study, with discrepancies resolved through discussion or consultation with a third reviewer if needed. Risk-of-bias assessments will be summarised descriptively and visually using traffic light plots and risk-of-bias summary figures.

Potential meta-biases, including publication bias and small-study effects, will also be examined. Funnel plots will be inspected for asymmetry for each primary outcome if at least 10 studies are available, and small-study effects will be statistically evaluated using Egger’s regression test for continuous outcomes and Peters’ test for binary outcomes.^34^ The inclusion of unpublished datasets in this IPDMA is expected to reduce the risk of publication bias by incorporating data that might not appear in peer-reviewed literature. To further evaluate the potential impact of meta-biases, sensitivity analyses will compare the results including all studies with analyses restricted to published studies only.

Finally, the overall confidence in the evidence will be informed by the pattern of risk-of-bias assessments, the consistency of effects across trials and the results of sensitivity analyses (eg, excluding high-risk-of-bias studies or published-only datasets).

## Data analysis

We will employ state-of-the-art Bayesian mixed-effects models to analyse participant-level data from all included trials using a one-stage IPDMA approach. This approach models all available data within a single hierarchical framework, preserving the clustering of participants within trials, maximising statistical power and enabling consistent handling of missing data.

Analyses will be conducted separately for the two outcome types: (1) child-related symptom severity and (2) clinician-rated clinical severity rating. Bayesian linear mixed-effects models will be used where change in dysfunctional cognitions from pre-treatment to post-treatment will be included as the primary predictor of follow-up outcome. A maximal random-effects structure will be specified, with random intercepts for trials and random slopes for key predictors.^38^ This approach accounts for both between- and within-study variability and allows more flexible modelling across heterogeneous samples. Between-study heterogeneity will be estimated through the variance components, with 95% credible intervals. All models will control for baseline levels of symptoms and dysfunctional cognitions.

Participant-level covariates such as age, gender, socioeconomic status, comorbidity and baseline cognitive distortions will be included as fixed effects or interaction terms to assess potential moderation. Trial-level covariates (eg, comparator type, treatment format, duration) may also be explored. Missing data will be handled using multiple imputation under the missing-at-random assumption, implemented directly within the Bayesian framework. Sensitivity analyses will assess the robustness of results, including models that exclude studies rated as high risk of bias and models incorporating risk-of-bias scores as trial-level covariates. Weakly informative priors will be used for regression coefficients. Model convergence will be assessed using R-hat statistics, trace plots and posterior predictive checks. All analyses will be conducted in R using appropriate Bayesian modelling packages (eg, *brms, rstanarm*), and analysis scripts and model code will be made openly available via an online repository following publication.

## Discussion

The present protocol describes the first IPDMA to investigate change in dysfunctional cognitions as a putative mechanism of action in CBT for youth with anxiety disorders, OCD and PTSD. This project will evaluate whether reductions in dysfunctional cognitions during treatment predict improvements in clinical severity and diagnostic remission at post-treatment and follow-up, and whether these associations vary by individual characteristics such as age, gender, socioeconomic status and comorbidity.

Previous efforts to examine changes in dysfunctional cognitions as a mechanism of CBT in youth have produced mixed findings,^23 24 26^ and evidence on individual differences remains limited due to small sample sizes in individual trials. By combining data across multiple international RCTs, this IPDMA will have sufficient statistical power to detect individual-level moderators and to evaluate whether changes in dysfunctional cognitions during treatment predict symptom improvement at post-treatment and follow-up. The IPDMA design enables consistent handling of missing data, harmonisation of outcome and predictor measures and robust modelling of associations across diverse clinical subgroups defined by age, gender, comorbidity and socioeconomic status.^34^ However, several limitations of this IPDMA must also be acknowledged. The study relies on secondary analysis of existing trial data, which may limit consistency in how key constructs, such as dysfunctional cognitions or socioeconomic status, were measured across studies. Harmonisation will be required, which may reduce measurement precision or lead to the exclusion of studies that do not meet minimum data requirements. Additionally, by averaging across different measures of dysfunctional cognitions to form a composite score, the analysis may obscure potentially meaningful differences between specific types of dysfunctional cognitions (eg, interpretation bias or negative self-statements). Future analyses may explore whether particular domains of cognitive dysfunction differentially predict treatment outcomes, where sufficient data are available. Another limitation pertains to the use of CSR, an ordinal scale derived from diagnostic interviews that will be analysed as a continuous variable. While this is a common approach in psychotherapy research, it assumes equal intervals between scale points and may introduce minor estimation bias in the analyses. Lastly, IPDMA cannot overcome limitations inherent to the original trials, such as variation in treatment fidelity, follow-up duration, or the timing of data assessments.^32 34^ Despite these challenges, this study has the potential to provide critical insights into the cognitive mechanisms underlying youth CBT. By clarifying whether and for whom changes in dysfunctional cognitions drive therapeutic improvements, this work may inform both research and clinical practice for children and adolescents with anxiety disorders, OCD and PTSD.

## Ethics and dissemination

This study involves secondary analysis of de-identified IPD obtained from randomised controlled trials. All data will be managed in compliance with the GDPR. All primary trials will have received ethical approval permitting data sharing and secondary use. The secondary analysis protocol has been approved by the Ethics Review Board of the University of Amsterdam (FMG-8972_2024). Findings will be disseminated through a peer-reviewed publication, presentations at national and international scientific conferences and open sharing of analysis scripts and harmonisation procedures.

## Supplementary material

10.1136/bmjopen-2025-113007 online supplemental file 1
