# Supplementary material for: Investigating dysfunctional cognition change as a putative mechanism of CBT for youth anxiety, OCD and PTSD: protocol for an individual participant data meta-analysis
Source: BMJ Open. 2025 Dec 3;15(12):e113007. doi: 10.1136/bmjopen-2025-113007 (PMC12682165; doi:10.1136/bmjopen-2025-113007)
Supplement: online supplemental file 1 [file bmjopen-15-12-s001.docx]

**Supplemental Material**

A systematic literature search was conducted in APA PsycINFO, MEDLINE, and Web of Science Core Collection in two rounds. The initial search yielded 1.063 results on 5 April 2024: PsycINFO (321 results), MEDLINE (364 results), and Web of Science Core Collection (378 results). The updated search on 8 September 2025 yielded 137 results: PsycINFO (42 results), MEDLINE (47 results), and Web of Science Core Collection (48 results). The search strategies, including limits and Boolean operators, are reported below for each database.

**PsycINFO (Ovid, APA PsycInfo)**

1. ((d#sfunct* OR distort* OR negativ* OR automatic*) ADJ (cogniti* OR interpretation* OR thinking OR thought*)).ti,ab,id.
2. anxiety disorders/ OR generalized anxiety disorder/ OR panic disorder/ OR phobias/ OR acrophobia/ OR agoraphobia/ OR claustrophobia/ OR ophidiophobia/ OR school phobia/ OR social phobia/ OR separation anxiety disorder/ OR obsessive compulsive disorder/ OR posttraumatic stress disorder/ OR complex ptsd/ OR posttraumatic stress/ OR (panic disorder* OR anxiety disorder* OR acrophobi* OR agoraphobi* OR arachnophobi* OR claustrophobi* OR ophidiophobi* OR phobi* OR obsessiv* compuls* OR OCD OR posttrauma* OR post-trauma* OR psychotrauma* OR psycho-trauma* OR PTSD* OR PTSS*).ti,ab,id.
3. (school age 6 12 yrs OR adolescence 13 17 yrs).ag. OR (child* OR pediat* OR paediat* OR kid OR kids OR preteen* OR teen* OR youngster* OR youth* OR minors* OR under ag* OR underag* OR juvenile* OR girl* OR boy* OR preadolesc* OR adolesc*).ti,ab,id.
4. 1 AND 2 AND 3

Key: / = subject heading, ti = title, ab = abstract, id = key concepts (other keywords added by PsycInfo indexers to supplement the subject headings), ag = age group, ADJ = words next to each other (in that specific order), * = unlimited amount of characters, # exactly 1 character

**MEDLINE (Ovid MEDLINE ALL, including Epub Ahead of Print, In-Process, In-Data-Review & Other Non-Indexed Citations and Daily)**

1. ((d#sfunct* OR distort* OR negativ* OR automatic*) ADJ (cogniti* OR interpretation* OR thinking OR thought*)).ti,ab,kf.
2. anxiety disorders/ OR panic disorder/ OR phobic disorders/ OR agoraphobia/ OR phobia, social/ OR obsessive-compulsive disorder/ OR stress disorders, post-traumatic/ OR (panic disorder* OR anxiety disorder* OR acrophobi* OR agoraphobi* OR arachnophobi* OR claustrophobi* OR ophidiophobi* OR phobi* OR obsessiv* compuls* OR OCD OR posttrauma* OR post-trauma* OR psychotrauma* OR psycho-trauma* OR PTSD* OR PTSS*).ti,ab,kf.
3. child/ OR adolescent/ OR (child* OR pediat* OR paediat* OR kid OR kids OR preteen* OR teen* OR youngster* OR youth* OR minors* OR under ag* OR underag* OR juvenile* OR girl* OR boy* OR preadolesc* OR adolesc*).ti,ab,kf.
4. 1 AND 2 AND 3

Key: / = medical subject heading (MeSH), ti = title, ab = abstract, kf = author supplied keywords, * = unlimited amount of characters, ADJ = words next to each other (in that specific order), # exactly 1 character

**Web of Science Core Collection (Web of Science Core Collection Editions: Science Citation Index Expanded (SCI-EXPANDED), Social Sciences Citation Index (SSCI), Arts & Humanities Citation Index (A&HCI), Emerging Sources Citation Index (ESCI)))**

1. TS=("d?sfunct* cogniti*" OR "distort* cogniti*" OR "negativ* cogniti*" OR "automatic* cogniti*" OR "d?sfunct* interpretation*" OR "distort* interpretation*" OR "negativ* interpretation*" OR "automatic* interpretation*" OR "d?sfunct* thinking" OR "distort* thinking" OR "negativ*thinking" OR "automatic* thinking" OR "d?sfunct* thought*" OR "distort* thought*" OR "negativ* thought*" OR "automatic* thought*")
2. TS=("panic disorder*" OR "anxiety disorder*" OR "acrophobi*" OR "agoraphobi*" OR "arachnophobi*" OR "claustrophobi*" OR "ophidiophobi*" OR "phobi*" OR "obsessiv* compuls*" OR "OCD" OR "posttrauma*" OR "post-trauma*" OR "psychotrauma*" OR "psycho-trauma*" OR "PTSD*" OR "PTSS*")
3. TS=("child*" OR "pediat*" OR "paediat*" OR "kid" OR "kids" OR "preteen*" OR "teen*" OR "youngster*" OR "youth*" OR "minors*" OR "under ag*" OR "underag*" OR "juvenile*" OR "girl*" OR "boy*" OR "preadolesc*" OR "adolesc*")
4. #1 AND #2 AND #3

Key: TS = topic, which includes title, abstract, author keywords and Web of Science Keywords Plus,* = unlimited number of characters, ? = exactly 1 character

**ISRCTN Registry (**[www.isrctn.com](https://www.isrctn.com)), **ClinicalTrials.gov (**[www.clinicaltrials.gov](https://www.clinicaltrials.gov)), **EU Clinical Trials Register (**[www.clinicaltrialsregister.eu](https://www.clinicaltrialsregister.eu)): ((child OR adolescent OR youth OR teen OR pediatric OR paediatric OR juvenile OR minor OR youngster OR preteen OR preadolescent) AND (anxiety OR "anxiety disorder" OR "panic disorder" OR agoraphobia OR phobia OR OCD OR "obsessive compulsive disorder" OR PTSD OR "post-traumatic stress disorder" OR "posttraumatic stress disorder") AND ("cognitive behavioural therapy" OR "cognitive behavioral therapy" OR CBT OR "exposure therapy" OR exposure) AND ("dysfunctional cognition" OR "dysfunctional thinking" OR "distorted cognition" OR "distorted thinking" OR "negative cognition" OR "negative thinking" OR "negative thoughts" OR "automatic thinking" OR "automatic thoughts" OR "maladaptive thinking" OR "cognitive distortion" OR "negative interpretation" OR "biased interpretation"))
